# British Society of Rheumatology guideline working group response to European Medicines Agency safety update on Hydroxychloroquine

**DOI:** 10.1093/rheumatology/kead384

**Published:** 2023-07-31

**Authors:** Mark D Russell, Mrinalini Dey, Julia Flint, Philippa Davie, Alexander Allen, Amy Crossley, Margreta Frishman, Mary Gayed, Kenneth Hodson, Munther Khamashta, Louise Moore, Sonia Panchal, Madeleine Piper, Clare Reid, Katherine Saxby, Karen Schreiber, Naz Senvar, Sofia Tosounidou, Maud van de Venne, Louise Warburton, David Williams, Chee-Seng Yee, Caroline Gordon, Ian Giles

**Affiliations:** Centre for Rheumatic Diseases, King’s College London, London, UK; Institute of Life Course and Medical Sciences, University of Liverpool, Liverpool, UK; Robert Jones and Agnes Hunt Orthopaedic Hospital NHS Foundation Trust, Shropshire, UK; Centre for Rheumatic Diseases, King’s College London, London, UK; British Society for Rheumatology, Clinical Affairs, London, UK; Patient Representative, London, UK; Queen's Hospital, Maternity Services, Barking Havering & Redbridge University NHS Trust, UK; Rheumatology, Sandwell and West Birmingham Hospital, UK; UK Tetralogy Information Service, London, UK; Department of Women & Children's Health, King's College London, London, UK; Rheumatic and Musculoskeletal Disease Unit, Our Lady’s Hospice and Care Service, Dublin, Ireland; Rheumatology, University Hospitals of Leicester, Leicester, UK; Royal National Hospital for Rheumatic Diseases, Royal United Hospital, Bath, UK; Patient Representative, London, UK; University College London Hospitals NHS Foundation Trust, Pharmacy, London, UK; Thrombosis and Haemostasis, Guy's and St Thomas' NHS Foundation Trust, London, UK; Danish Hospital for Rheumatic Diseases, Sonderborg, Denmark; Department of Regional Health Research (IRS), University of Southern Denmark, Odense, Denmark; Obstetrics and Gynaecology, St George's University Hospitals NHS Foundation Trust, London, UK; Lupus UK Centre of Excellence, Sandwell and West Birmingham Hospitals NHS Trust, Birmingham, UK; Obstetrics & Gynaecology, Frimley Park Hospital, Surrey, UK; Primary Care and Health Sciences, Keele University, Keele, UK; Womens Health, University College London Hospitals NHS Foundation Trust, London, UK; Department of Rheumatology, Doncaster and Bassetlaw Teaching Hospitals NHS Foundation Trust, Doncaster, UK; Rheumatology Research Group, Institute of Inflammation and Ageing, University of Birmingham, Birmingham, UK; Centre for Rheumatology, Division of Medicine, University College London, London, UK

Rheumatology key message

- Reassurance that evidence underlying the EMA safety alert on HCQ in pregnancy does not alter the BSR 2022 pregnancy recommendations.

Dear Editor, We are concerned by the European Medicines Agency (EMA) recommendation to update the patient information leaflet for the use of HCQ in pregnancy [1]. HCQ is the antimalarial drug most used to treat rheumatic diseases. It has been extensively studied in pregnancy, and the British Society of Rheumatology guidelines on prescribing antirheumatic drugs in pregnancy recommend its use in pregnancy if required to treat disease at doses up to 400 mg/day [2].

The EMA recommendations from pharmacovigilance monitoring were influenced by a population-based cohort study comparing HCQ-exposed (*n* = 2045) and HCQ-unexposed (*n* = 21 679) rheumatic disease pregnancies [3]. It found a small increase in risk of congenital malformations from 35.3/1000 in women not taking HCQ to 44.1/1000 in those taking HCQ in the first trimester. Moreover, a statistically significant increase in risk was found only with daily doses ≥400 mg of HCQ and no comparison was made between HCQ exposure at typical rheumatology dosing of up to and including 400 mg/day compared with atypical dosing of >400 mg/day.

The patient information leaflet will be revised to describe findings solely from this study and remove mention of other studies with reassuring findings. The revised wording will be: ‘[Hydroxychloroquine] may be associated with a small increased risk of major malformations and should not be used during pregnancy unless your doctor considers the benefits outweigh the risks.’ The summary of product characteristics recommendation is unchanged from: ‘Hydroxychloroquine should be avoided in pregnancy except when, in the judgement of the physician, the individual potential benefits outweigh the potential hazards. If treatment with hydroxychloroquine is necessary during pregnancy, the lowest effective dose should be used.’

Safety alerts on medication use in pregnancy have far-reaching consequences on prescribing of drugs used by rheumatologists. In 2019 the EMA [4] and Medicines and Healthcare products Regulatory Agency (MHRA) [5] issued warnings that ondansetron should not be used to treat nausea of pregnancy in the first trimester due to an increased risk of orofacial malformations, specifically oral clefts. These warnings were based on findings that showed a small but statistically significant increase in risk [6], with the absolute increased risk of oral clefts from ondansetron being 3 extra cases per 10 000 women treated. Consequently, healthcare professionals and patients were deterred from use of this drug in pregnancy. As the substantial suffering from hyperemesis gravidarum not infrequently results in termination of pregnancy, expert opinion remains that ondansetron is an effective low-risk treatment of this condition that should be an option to the informed pregnant woman [7].

The Huybrechts study on HCQ was included in the systematic literature review that informed the 2022 BSR pregnancy guideline based on 43 studies of 4701 pregnancy exposures to HCQ. There were no appreciable adverse effects overall of HCQ on pregnancy duration or birth weight, no increased risk of first trimester miscarriages and no specific patterns of congenital malformations in association with HCQ exposure [2].

We would like to reassure healthcare professionals and patients that the evidence under-pinning the EMA safety alert on use of HCQ in pregnancy was considered and does not alter the BSR 2022 pregnancy recommendations. Other examples of regulatory bodies drawing different conclusions from the same data exist within the BSR guideline. The MHRA and EMA have issued guidance for infants exposed to infliximab (a TNF inhibitor) *in utero* [8]. They recommend that infants exposed to infliximab *in utero* should not receive live vaccinations until 12 months of age and that live vaccinations should be avoided in infants exposed to infliximab through breast milk. The systematic review of the same body of evidence led the multi-disciplinary authors of the updated BSR pregnancy guidelines to recommend avoidance of live vaccine use in infants exposed to all TNF inhibitor and non-TNF inhibitor biologic DMARDs with high rates of placental passage, in third trimester of pregnancy until they are 6 months of age [2]. Other, experts have cautioned against a one size fits all approach and suggest that obstetric teams continue to provide well-considered and evidence-based advice on effects of maternal biologic DMARDs on suitability of childhood vaccinations [9].

Our multidisciplinary working group is concerned that a statement emphasizing a possible small risk of harm over established benefit may encourage healthcare professionals and patients to stop HCQ in pregnancy and risk disease relapse to the detriment of mother and fetus. A more supportive wording would acknowledge the potential for a small increased risk of major congenital malformations at doses higher than those normally used in rheumatological conditions and state ‘the benefits and risks of HCQ during pregnancy should be considered with your specialist before conception’. It would therefore encourage consultations with specialists to balance the benefits of HCQ to prevent maternal disease flare and harm to the baby that may occur if this drug is stopped in pregnancy against potential small risks of congenital malformations at high dose.

## Data Availability

Not applicable for this letter.
